# XRCC3 Thr241Met Polymorphism and Clinical Outcomes of NSCLC Patients Receiving Platinum-Based Chemotherapy: A Systematic Review and Meta-Analysis

**DOI:** 10.1371/journal.pone.0069553

**Published:** 2013-08-05

**Authors:** Xiao-yong Shen, Fan-zhen Lu, Yun Wu, Li-ting Zhao, Zhi-feng Lin

**Affiliations:** 1 Department of Thoracic Surgery, The Huadong Hospital, Shanghai Fudan University, Shanghai, China; 2 Department of Thoracic Surgery, Shanghai First Peoples' Hospital, School of Medicine, Shanghai Jiao Tong University, Shanghai, China; MOE Key Laboratory of Environment and Health, School of Public Health, Tongji Medical College, Huazhong University of Science and Technology, China

## Abstract

**Introduction:**

X-ray repair cross-complementing protein 3 (XRCC3) is an essential gene involved in the double-strand break repair pathway. Published evidence has shown controversial results about the relationship between XRCC3 Thr241Met polymorphism and clinical outcomes of non-small cell lung cancer (NSCLC) patients receiving platinum-based chemotherapy.

**Methods:**

A systematic review and meta-analysis was performed to evaluate the predictive value of XRCC3 Thr241Met polymorphism on clinical outcomes of advanced NSCLC receiving platinum-based chemotherapy. Response to chemotherapy, overall survival (OS) and progression-free survival (PFS) were analyzed.

**Results:**

A number of 11 eligible studies were identified according to the inclusion criteria. Carriers of the variant XRCC3 241Met allele were significantly associated with good response to platinum-based chemotherapy (ThrMet/MetMet vs. ThrThr: OR  = 1.509, 95% CI: 1.099–2.072, P_heterogeneity_  = 0.618). The XRCC3 Thr241Met polymorphism was not associated with OS (MetMet vs. ThrThr, HR  = 0.939, 95% CI:0.651–1.356, P_heterogeneity_  = 0.112) or PFS (MetMet vs. ThrThr, HR  = 0.960, 95% CI: 0.539–1.710, P_heterogeneity_  = 0.198). Additionally, no evidence of publication bias was observed.

**Conclusions:**

This systematic review and meta-analysis shows that carriers of the XRCC3 241Met allele are associated with good response to platinum-based chemotherapy in advanced NSCLC, while the XRCC3 Thr241Met polymorphism is not associated with OS or PFS.

## Introduction

Lung cancer is the major public health problem worldwide, which causes more than one million deaths each year [1]. About 80% of newly diagnosed lung cancer cases are non-small cell lung cancer (NSCLC), and most of them are at advanced stage [2]. Currently, the prognosis for NSCLC is still poor, with a 5-year survival rate less than 15% [3]. Platinum-based chemotherapy is the main treatment of choice for advanced NSCLC. However, evidence suggests that the efficacy of platinum-based chemotherapy varies remarkably among individuals with NSCLC, with a response rate of 26%–60% [4].

Platinum agents are known to function through the formation of DNA adducts that inhibit DNA synthesis and transcription. Proposed mechanisms of resistance to platinum-based chemotherapy include inactivation of platinum compounds through the glutathione metabolic pathway and increased tolerance to DNA damage as a consequence of enhanced DNA repair capacity. Previous studies have demonstrated that single nucleotide polymorphisms (SNPs) influence DNA repair activity and removal of DNA adducts [5]–[7]. Molecular epidemiology studies have documented that lung cancer risk [8], [9], DNA repair capacity and levels of DNA damage [10] may be modulated by SNPs of genes in the DNA repair pathways like, double-strand break (DSB) repair, base excision repair (BER) and nucleotide excision repair (NER). X-ray repair cross-complementing protein 3 (XRCC3) plays a key role in DSB repair during homologous recombination, which is essential for maintaining chromosome stability [11]. It has been reported that the functional SNP in codon 241(Thr to Met, rs861539 C>T) of XRCC3 is associated with the level of bulky DNA adducts [12], sensitivity to chemotherapy and risk of lung cancer [9]. A number of studies have investigated the association of XRCC3 Thr241Met polymorphism with clinical outcomes of NSCLC receiving platinum-based chemotherapy [13]–[16]; however, the results were quite controversial and inconsistent.

In this systematic review and meta-analysis, we comprehensively evaluated the correlation between XRCC3 Thr241Met polymorphism and clinical outcomes (response rate, overall survival and progression-free survival) of advanced NSCLC patients receiving platinum-based chemotherapy.

## Patients and Methods

### Identification of Eligible studies

Online databases of PubMed and EMBASE were searched to retrieve potentially relevant studies. A comprehensive search were conducted using combination of key words and medical subheadings of “X-ray repair cross complementing protein 3” or “XRCC3”, “single nucleotide polymorphism” or “SNP” or “polymorphism”, and “lung cancer” or “lung tumor” or “neoplasm, lung”. There was no limitation of research and the latest research was performed on April 2013. References of related studies and reviews were manually searched for additional studies.

### Inclusion and exclusion criteria

Records retrieved were primarily screened by titles and abstracts. Then, potentially relevant full-text articles were retrieved and further assessed for eligibility. Eligible studies were identified according to the following criteria: 1) patients with NSCLC and treated with platinum-based chemotherapy; 2) investigating the association between XRCC3 Thr241Met polymorphism and clinical outcomes, i.e. response, overall survival (OS) and progression free survival (PFS); 3) published studies with available full-text articles. All records were screened by two investigators independently (SXY and LFZ) with disagreement resolved by discussion.

### Data extraction and methodological quality assessment

Data from eligible studies was collected independently by two investigators (SXY and LFZ) using a standard data collection form and they reached consensus on each item. The following data was extracted: first author, year of publication, the country where the study conducted, age, male, ethnicity, number of patients, TNM stage, chemotherapy regimens, genotype distribution among responders and non-responders, hazard ratios (HR) and corresponding 95% confidence intervals (CI) for OS and PFS. Ethnicity was simply classified as Asian, Caucasian and Mixed. Response to chemotherapy was assessed with RECIST criteria [17], while “good response” was defined as complete response + partial response and “poor response” was stable disease + progressive disease. In one study reported by de las Peñas [11], HR and 95% CIs for OS were estimated from Kaplan-Meier curves according to the methods introduced by Tierney JF [18].

### Statistical analysis

The association strength of XRCC3 Thr241Met polymorphism with response to platinum-based chemotherapy was estimated with odds ratio (OR) and 95% CIs in 4 comparison model: homozygote comparison (MetMet vs. ThrThr), heterozygote comparison [ThrMet vs. ThrThr], dominant model (ThrMet/MetMet vs. ThrThr) and recessive model (MetMet vs. ThrMet/ThrThr), assuming the dominant and recessive effect of the Met allele, respectively). For OS and PFS, the pooled HR and 95% CIs were calculated with HRs and 95% CIs extracted from eligible studies and only homozygote comparison and heterozygote comparison were performed.

Heterogeneity between studies was detected using chi-square by Q test, and p<0.1 indicated significant heterogeneity [19]. For response, the pooled OR and 95% CIs were calculated with fixed-effects model in the absence of significant heterogeneity or random-effects model in the presence of heterogeneity. For OS and PFS, all data synthesis was conducted using random-effects model owing to limited number of studies. Sub-group analyses according to ethnicities were performed. Publication bias was tested via Begg's funnel plot and the Egger's linear regression test, and a p<0.05 was considered significant [20]. Sensitivity analysis was performed to evaluate the influence of individual studies' on the pooled result. All statistical analyses were calculated with STATA software (version 10.0; StataCorp, College Station, Texas USA). And all P values are two-side.

## Results

**Figure 1:**
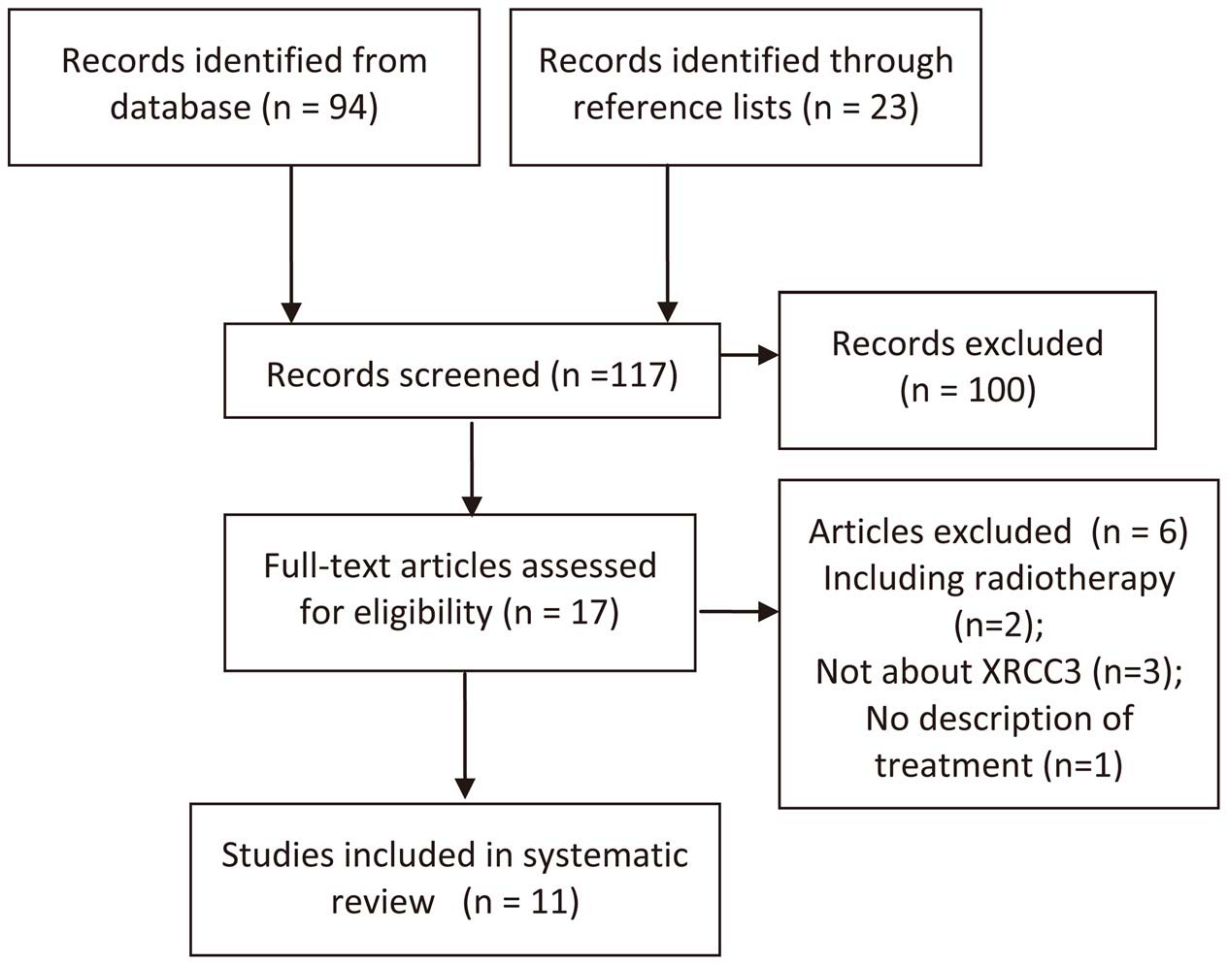
Flow Diagram.

The detailed process of study identification was shown in Figure 1. A number of 117 records were screened and 6 full-text articles were excluded for the reason of including patients received radiotherapy [21], [22], not about XRCC3 [23]–[25] and no description of treatment [26]. Overall, 11 [7], [11], [13]–[16], [27]–[31] eligible studies were identified and 9 [7], [11], [13]–[16], [27]–[29] were included in meta-analysis. The baseline characteristics of 11 studies were shown in Table 1. In total, 2201 patients with advanced NSCLC were enrolled (one study [16] included NSCLC with stage of I–IV, but most of them were at advanced stage, Table 1). Cisplatin and carboplatin were the most common platinum drugs. Most studies were carried out among Asian and Caucasian patients.

**Table 1:** Baseline Characteristics of Eligible Studies.

Author	Year	Country	Ethnicity	Chemotherapy	TNM Stage	Num	Age a	Male
Provencio M	2012	Spain	Caucasian	cisplatin+vinorelbine	IIIB –IV	180	62(39–78)	87.20%
Ke HG	2012	China	Asian	cisplatin+gemcitabine/docetaxel/ vinorelbine/paclitaxel	III –IV	460	59.5±3.5	72.60%
Liao WY b	2012	China	Asian	gemcitabine/bevacizumab+cisplatin /carboplatin/oxaliplatin	IIIB–IV	62	57(36–78)	56.50%
Chen X	2011	China	Asian	cisplatin/carboplatin+gemcitabine/ vinorelbine/paclitaxel/docetaxel	IIIB–IV	355	60(32–78)	69.90%
Xu C	2011	China	Asian	cisplatin+gemcitabine/docetaxel/ vinorelbine/paclitaxel	IIIB–IV c	130	62(28–83)	69.20%
Ludovini V	2011	Italy	Caucasian	cisplatin+gemcitabine/vinorelbine/ taxol or gemcitabine alone	IIIB –IV	192	63(25–81)	74.00%
Joerger M	2011	Netherlands	Mixed	gemcitabine+cisplatin/carboplatin	IIIB –IV	137	59.7(37–79)	56%
Zhou C	2010	China	Asian	cisplatin+gemcitabine/vinorelbine /paclitaxel	IIIB –IV	130	61(30–78)	56.90%
de las Peñas R	2005	Italy	Caucasian	cisplatin+gemcitabine	IIIB –IV	135	62 (31–81)	92.60%
Metro G d	2011	Italy	Caucasian	gemcitabine/paclitaxel/etoposide/ + cisplatin/carboplatin	IIIB –IV	80	59(26–78)	61.30%
Ren S d	2011	China	Asian	cisplatin+gemcitabine/docetaxel/ vinorelbine/paclitaxel	IIIB –IV	340	60(30–78)	68.20%

a: age is presented as median and range or mean + standard deviation; b: data was only extracted from the training set; c: percentage not available; d: studies not included in quantitative synthesis.

### Response to platinum-based chemotherapy

**Figure 2:**
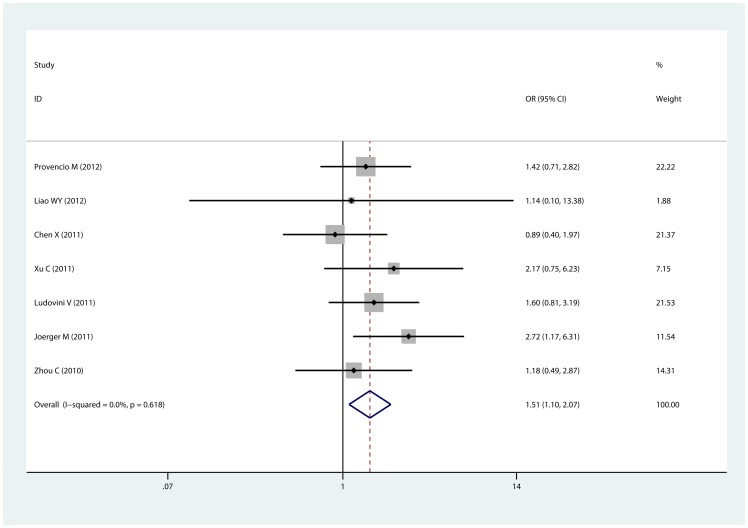
Association of XRCC3 Thr241Met polymorphism with response to platinum-based chemotherapy. The forest plot is generated from the comparison of ThrMet+MetMet vs. ThrThr.

**Figure 3:**
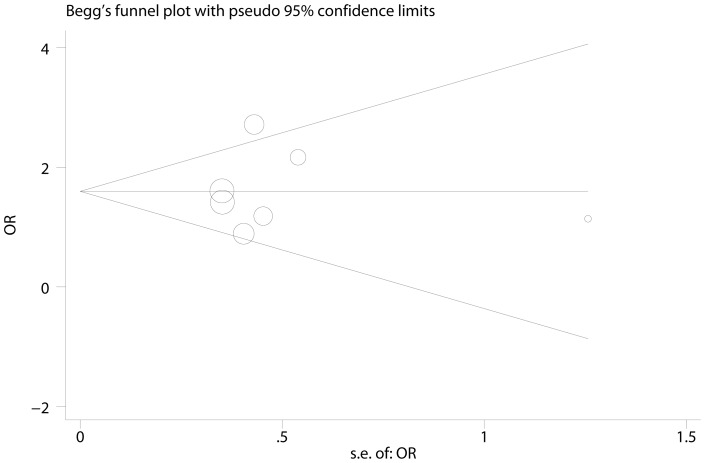
Funnel plot for the dominant model of XRCC3 Thr241Met polymorphism and response to platinum-based chemotherapy. No evidence of publication bias was found (P_Egger_  = 0.907, P_Begg_  = 1).

XRCC3 Thr241Met polymorphism and response to platinum-based chemotherapy was investigated in 8 [7], [13]–[15], [27]–[29], [31] studies and 7 [7], [13]–[15], [27]–[29] of them were included in meta-analysis. The chemotherapy regimens in eligible studies were comparable (Table 1). By pooling available data, we found that the variant Met allele was significantly associated with good response to chemotherapy in three comparison models (for example dominant model, ThrMet/MetMet vs. ThrThr: OR  = 1.509, 95% CI: 1.099–2.072, Pheterogeneity  = 0.618; Table 2, Figure 2). In sub-group analyses, the mutant Met allele indicated good response in the sub-group of Caucasian and Mixed population, but not Asian. Notably, no significant heterogeneity was observed. To evaluate the influence of individual studies, sensitivity analysis was performed and the result suggests that no individual study affected the pooled result significantly (figure not shown). Egger's test and Begg's test were performed to detect publication bias and no evidence of publication bias was detected (P_Egger_  = 0.907, P_Begg_  = 1; Figure 3). Sensitivity analysis and detection of publication bias were based on dominant model in that 7 studies were available for this comparison.

**Table 2:** XRCC3 Thr241Met polymorphism and response to platinum-based chemotherapy.

	Homozygote Comparison			Heterozygote Comparison			Recessive Model			Dominant Model		
	Study	OR(95% CI)	P	Study	OR(95% CI)	P	Study	OR(95% CI)	P	Study	OR(95% CI)	P
Overall	3	1.983 (1.092–3.599)*	0.868	5	1.744 (1.169–2.601)*	0.527	3	1.390 (0.839–2.303)	0.343	7	1.509 (1.099–2.072)*	0.618
Ethnicity												
Asian		NA	NA	2	1.953 (0.745–5.120)	0.875		NA	NA	4	1.197 (0.728–1.969)	0.803
Caucasian	2	2.148 (1.073–4.298)*	0.78	2	1.349 (0.807–2.254)	0.638	2	1.751 (0.967–3.172)	0.695	2	1.509 (0.928–2.455)	0.625
Mixed	1	1.569 (0.483–5.098)*	NA	1	3.243 (1.349–7.795)*	NA	1	0.755 (0.275–2.075)	NA	1	1.509 (1.099–2.072)*	NA

* significant difference; Homozygote Comparison: MetMet vs. ThrThr; Heterozygote Comparison: ThrMet vs. ThrThr; Recessive Model: MetMet vs. ThrMet/ThrThr; Dominant Model: ThrMet/MetMet vs. ThrThr; OR: odds ratio; CI: confidence intervals; P: p value of heterogeneity; NA: not available.

### Overall Survival and Progression-Free Survival

**Figure 4:**
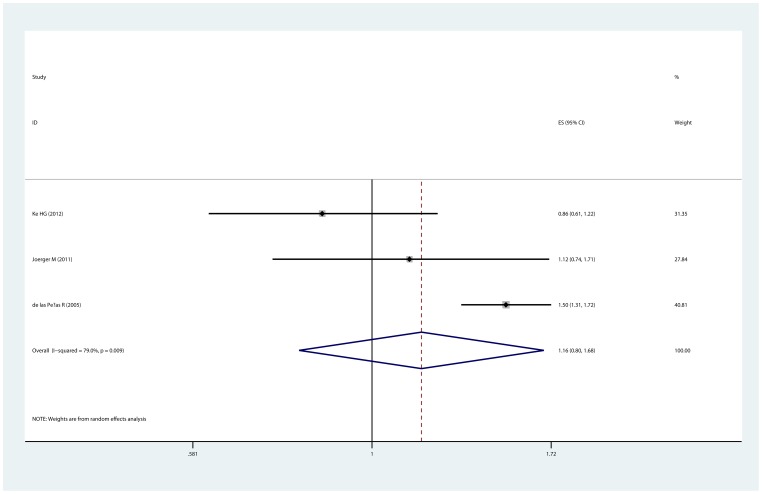
Association of XRCC3 Thr241Met polymorphism with overall survival. The forest plot is generated from the comparison of ThrMet vs. ThrThr.

Survival of advanced NSCLC and XRCC3 Thr241Met polymorphism was reported in 6 studies [11], [13]–[15], [30], but only 4 [11], [13]–[15] studies provided available data for meta-analysis. Indirect HR for OS was estimated from Kaplan-Meier curve in one study [11]. The median follow-up time for each study was 8.6 months [15], 9.7 months [11], 28.7 months [14] and 40 months [13]. For OS, no significant association between Thr241Met polymorphism and survival was observed (MetMet vs. ThrThr, OR  = 0.939, 95% CI:0.651–1.356, P_heterogeneity_  = 0.112; ThrMet vs. ThrThr, OR  = 1.162, 95% CI:0.802–1.683, P_heterogeneity_  = 0.009, Figure 4). Additionally, there was no significant correlation between Thr241Met polymorphism and PFS (MetMet vs. ThrThr, OR  = 0.960, 95% CI: 0.539–1.710, P_heterogeneity_  = 0.198; ThrMet vs. ThrThr, OR  = 0.831, 95% CI: 0.642–1.075, P_heterogeneity_  = 0. 927). Since the number of studies in the analyses of OS and PFS was too little, sensitivity analysis, Egger's test and Begg's test were not performed.

## Discussion

In this meta-analysis, we provided evidence that the XRCC3 241Met allele indicated good response to platinum-based chemotherapy, while the XRCC3 Thr241Met polymorphism was not associated with OS or PFS.

Platinum compounds, such as cisplatin and carboplatin, are activated intracellularly, binding to DNA to form bulky DNA adducts, block DNA replication and lead to cell death ultimately. Previous studies have suggested that suboptimal DNA repair capacity may lead to decreased removal of platinum-DNA adducts and favorable clinical outcomes [5], [32]. Functional SNPs occurring in the key genes of DNA repair pathways can alter DNA repair capacity [33] [34]) and various studies have evaluated the potentially predictive value of these SNPs [32], [35], [36]. In addition to SNPs of individual genes, current studies have also investigated the whole DNA repair pathway [10], [37], [38]. For example, the predictive value of XRCC1 Arg399Gln polymorphism has been validated [39].

It has been documented that XRCC3 is integral to DNA DSB repair and the Thr241Met polymorphism has been associated with the level of bulky DNA adducts [12]. Individuals with the XRCC3 MetMet genotype had higher levels of DNA adducts, regardless of smoking status. Previous clinical studies have evaluated the predictive value of XRCC3 Thr241Met polymorphism as a biomarker. While these studies were of small size and under-powered to detect significant difference. In the present systematic review and meta-analysis, by including 9 studies and 2201 patients for quantitative synthesis, we found the Met allele was associated with a higher response rate to platinum-based chemotherapy, which is in agreement with molecular epidemiology data.

For survival of NSCLC patients, we found no association of XRCC3 Thr241Met polymorphism with OS or PFS. However, de las Peñas and colleagues [11] showed that, compared with ThrThr genotype, patients with the MetMet genotype were associated with a significantly longer survival in the sub-group of cisplatin-gmecitabine, which was also observed by Chen X [14]. Additionally, Chen X et al found that this association is chemotherapy-specific. In the sub-population of non-gemcitabine, the OS in patients with genotype of XRCC3 ThrThr was significantly longer than those with ThrMet or MetMet. By pooling all available data, we did not find any association of XRCC3 Thr241Met polymorphism with OS or PFS. Limited by number of studies, sub-group analyses according to chemotherapy regimens were not permuted and we failed to clarify the chemotherapy-specific association. Although, it is supposed that the XRCC3 Thr241Met polymorphism may be relevant to pharmacogenomics changes of gemcitabine and other anti-microtubule drugs.

In this systematic review and meta-analysis, we identified 11 eligible studies and 2201 NSCLC patients and provided a systematic overview of current studies. No significant heterogeneity was observed in the process of quantitative synthesis. Additionally, results of sensitivity analysis, Egger's test and Begg's test also confer the reliability and stability of our results. However, limitation to this meta-analysis should be noted. First, the number of studies was relatively small in that sub-group analyses were not available to explore the effect of chemotherapy regimens. Second, the association strength of XRCC3 Thr241Met and survival was based on small number of studies. Third, our results were based on raw data and was not adjusted for certain confounding factors, such as gender, age, TNM stage, histology type of NSCLC and chemotherapy regimens.

To summary, results of this systematic review and meta-analysis suggest that, in patients with advanced NSCLC, carriers of the XRCC3 241Met polymorphism is associated with good response to platinum-based chemotherapy. While the XRCC3 Thr241Met polymorphism is not associated with OS or PFS. However, future studies with large sample sizes and well designs are warranted to confirm these findings.

## Supporting Information

Table S1 PRISMA checklist.
(DOC)
